# Assessing the impact of three feeding stages on rumen bacterial community and physiological characteristics of Japanese Black cattle

**DOI:** 10.1038/s41598-024-55539-y

**Published:** 2024-02-28

**Authors:** Huseong Lee, Minji Kim, Tatsunori Masaki, Kentaro Ikuta, Eiji Iwamoto, Koki Nishihara, Itoko Nonaka, Akane Ashihara, Youlchang Baek, Sungdae Lee, Yoshinobu Uemoto, Satoshi Haga, Fuminori Terada, Sanggun Roh

**Affiliations:** 1 Graduate School of Agricultural Science, Tohoku University, Sendai, 980-8572 Japan; 2 National Institute of Livestock and Grassland Science, National Agriculture and Food Research Organization, Ikenodai, Tsukuba 305-0901 Japan; 3 Hyogo Prefectural Technology Center of Agriculture, Forestry and Fisheries, Kasai, Hyogo 679-0198 Japan; 4 Animal Nutrition and Physiology Division, National Institute of Animal Science, Wanju, 55365 South Korea

**Keywords:** Metabolomics, Animal physiology, Microbial communities

## Abstract

In Japan, Japanese Black cattle, known for their exceptional meat quality owing to their abundant intramuscular fat, undergo a unique three-stage feeding system with varying concentrate ratios. There is limited research on physiological and rumen microbial changes in Japanese Black cattle during these stages. Therefore, this study aimed to examine Japanese Black steers in these three stages: early (T1, 12–14 months), middle (T2, 15–22 months), and late (T3, 23–30 months). The rumen bacteria of 21 cattle per phase was analyzed using 16S rRNA gene sequencing. Rumen bacterial diversity was significantly higher in T1, with a distinct distribution, than in T2 and T3. Specific phyla and genera were exclusive to each stage, reflecting the shifts in feed composition. Certain genera dominated each stage: T1 had *Flexilinea*, *Streptococcus*, *Butyrivibrio*, *Selenomonas*, and *Kandleria*; T2 had *Bifidobacterium*, *Shuttleworthia*, and *Sharpea*; and T3 had *Acetitomaculum*, *Mycoplasma*, *Atopobium*, and *Howardella*. Correlation analysis revealed significant associations between certain microbial populations and physiological parameters. These findings indicate that changes in energy content and feed composition are associated with physiological and ruminal alterations. This study may guide strategies to improve rumen health and productivity in Japanese Black cattle by modifying diets to specific fattening stages.

## Introduction

Ruminants host diverse microbiota in their rumen, having symbiotic relationship with bacteria, archaea, viruses, fungi, and protozoa. Certain ruminal microbes significantly influence feed efficiency, nitrogen digestibility, and methane generation in ruminants^1–4^. The composition and function of the rumen microbiome are associated with economically valuable traits in cattle; hence, understanding the rumen microbiome can help identify and use causal relationships for trait improvement in livestock production^5,6^. Furthermore, understanding rumen microbial function has several advantages because the nutritional supply of the host animal is largely controlled by rumen dynamics.

The Japanese Black cattle breed is well-known for its exceptional intramuscular fat deposition and is raised to produce high-quality meat. Marbling refers to the degree of fat within the muscle and is considered the most important trait for determining carcass value in Japan^7^. Therefore, Japan has developed a distinct feeding management system that involves using high amounts of concentrate during the fattening period to enhance fat deposition. Japanese Black cattle have different physiological characteristics compared to other beef cattle. In our previous research^8^, we found that physiological changes during the fattening phase were influenced by high-energy feeds for growth and carcass traits in Japanese Black steers. Liver transcriptome analysis revealed that fat-related metabolism was activated during the fattening period. Rumen microbes are important for the growth and physiological performance of animals. Alternations in feed compositions can impact the population of the rumen microbes, which in turn affects the fermentation and digestion of feed, ultimately influencing the performance of animals in response to physiological changes. However, there is a lack of research on the correlation between physiological characteristics and rumen fermentation in this cattle breed, particularly in the context of rumen microbiota. Therefore, it is crucial to examine the rumen microbiota of Japanese Black cattle raised under different feeding management practices developed in Japan.

Multiple factors contribute to the variation in rumen microbiota, and breed per se significantly impacts the composition of cattle ruminal microbiota^9–11^. Recently, there has been ongoing analysis of the rumen microbiome in Japanese Black cattle^12,13^, but discussions have mainly focused on the relationship between rumen fermentation characteristics and microorganisms. Moreover, blood metabolites originate from the metabolites produced by rumen microbes; therefore, analyzing the connection between the rumen microorganisms influencing rumen fermentation and the blood metabolites reflecting the outcomes of rumen fermentation would be useful. We hypothesize that the differences in feed composition at each fattening stage will result in the formation of different rumen bacterial community. Hence, the relationship between rumen microbes, rumen fermentation, and blood metabolites will be altered. Therefore, this study aimed to reveal the relation between rumen microbiota and physiological features during the fattening period of Japanese Black cattle. To gain a comprehensive understanding of the metabolic profiles of Japanese Black cattle during their fattening period, we collected extensive rumen microbiome data and identified the core rumen microbiome related to physiological indicators.

## Results

### Rumen microbiota composition

**Figure 1:**
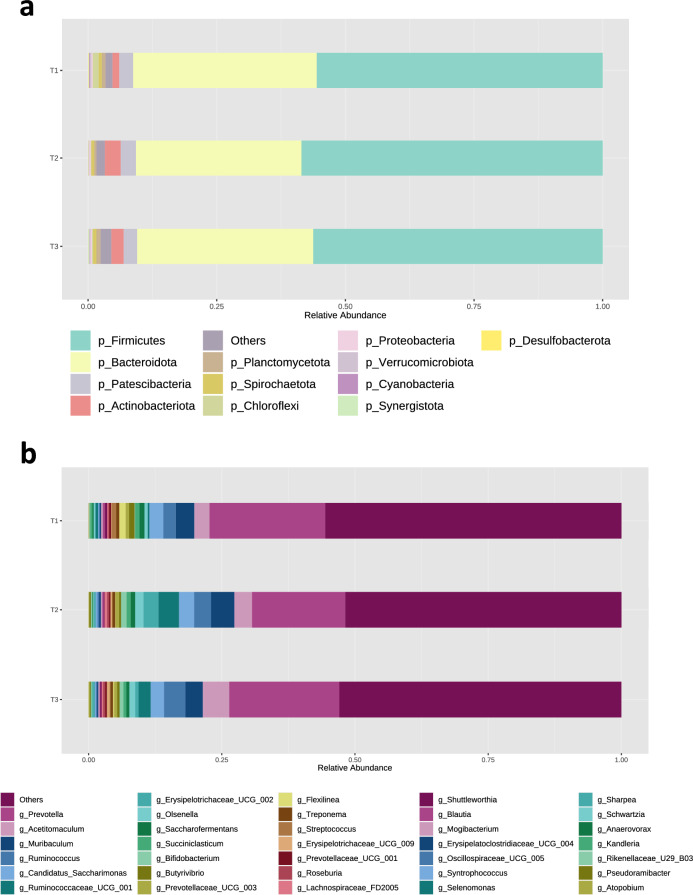
Composition of rumen microbiota in Japanese Black cattle. The relative abundances of (**a**) group average of phyla and (**b**) group average of genera are visualized. Only taxa with a percentage relative abundance of > 0.05% and prevalence of at least 20% in 62 Japanese Black cattle are shown and taxa accounting for < 0.05% of all sequences and prevalence of < 20% are included in “Others.” *T1* early fattening period, *T2* middle fattening period, *T3* late fattening period, *p_* phyla, *g_* genus.

The sequencing of bacterial 16S rRNA genes in the rumen fluid of Japanese Black cattle produced 2,844,321 raw sequence reads, with an average of 45,876 ± 12,017 reads per sample. An overview of the Illumina MiSeq sequenced datasets is provided in Supplementary table 1. Taxonomic entities that comprised at least 0.05% relative abundance and 20% prevalence of the overall sequences across all 62 rumen samples were considered "major taxa" and analyzed further. Firmicutes were the most prevalent, representing 56.8% of all sequences, followed by Bacteroidota, accounting for 34.1% of all sequences. Patescibacteria represented 2.7% of the total sequences, followed by Actinobacteriota (2.2%) and Planctomycetota (0.6%) (Fig. 1a). At the genus level, *Prevotella* was the most dominant, constituting 20.0% of all sequences observed across the 62 samples, followed by *Acetitomaculum* (3.8%), *Muribaculum* (3.7%), *Ruminococcus* (3.2%), *Candidatus* Saccharimonas (2.7%), *Ruminococcaceae* *UCG-001* (2.1%), *Erysipelotrichaceae* *UCG-002* (1.1%), *Olsenella* (1.1%), *Saccharofermentans* (0.8%), *Succiniclasticum* (0.7%), *Bifidobacterium* (0.6%), *Butyrivibrio* (0.6%), *Prevotellaceae* *UCG-003* (0.6%), *Flexilinea* (0.5%), and *Treponema* (0.5%) (Fig. 1b). The composition of the rumen microbiota in individual cattle is presented in Supplementary Fig. 3.

### Quantification of rumen microbiome and differentially abundant rumen microbial taxa

**Figure 2:**
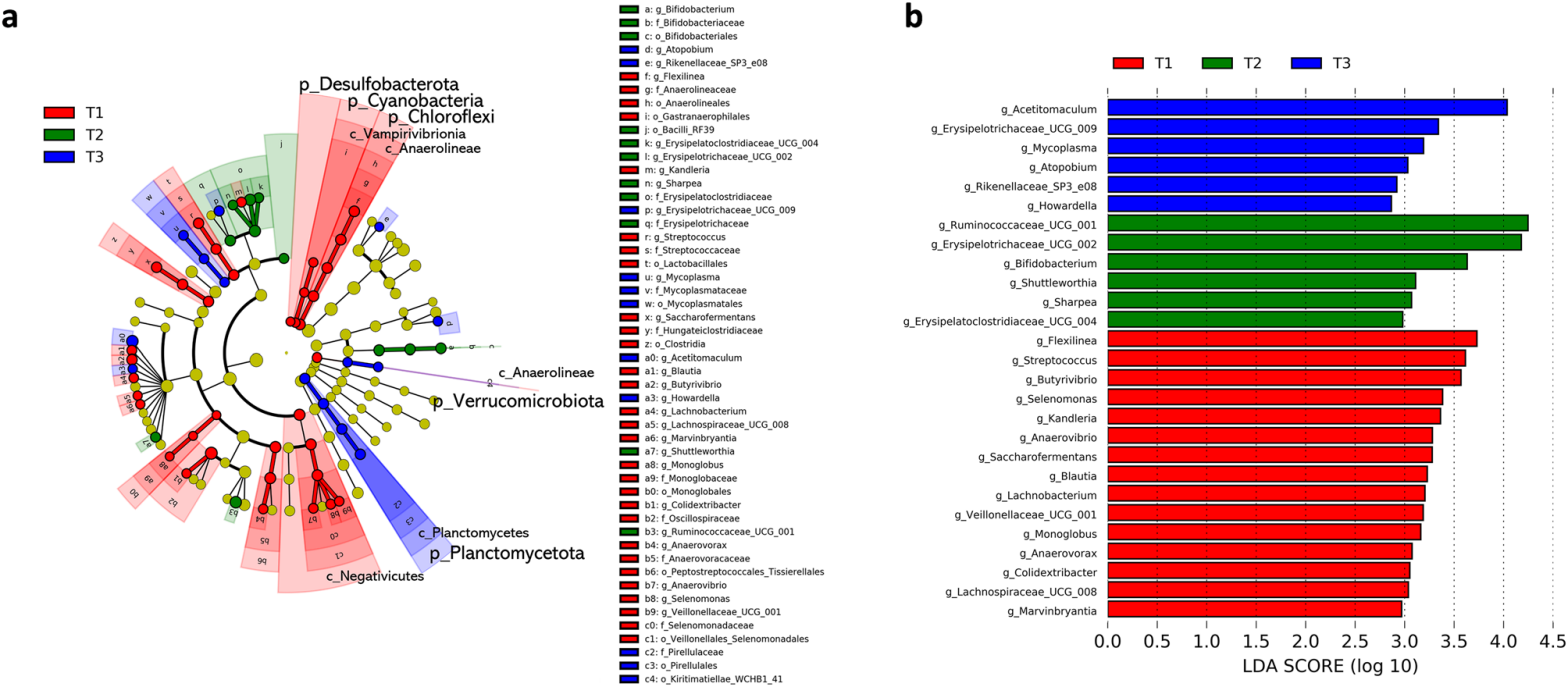
Differentially abundant multi-kingdom rumen microbiota among the three fattening periods. Major multi-kingdom (relative abundance > 0.05% and prevalence > 20%) that were differentially abundant among the three fattening period groups were detected using LEfSe with an LDA score of ≥ 2 and visualized using cladograms (**a**). Only genera are visualized using bar graph (**b**). *T1* early fattening period, *T2* middle fattening period, *T3* late fattening period, *p* phyla, *c* class, *o* order, *f* family, *g* genera.

Quantification of the gene copies of total bacteria, fungi, and ciliate protozoa is shown in Supplementary Fig. 1. The populations of total bacteria, fungi, and ciliate protozoa in the late fattening state (T3) were significantly lower than those in the early fattening stage (T1; P < 0.05). The present study utilized linear discriminant analysis (LDA) effect size (LEfSe) for statistical analyses, which identified microbial differences at phylum to genus levels during the fattening period (Fig. 2). At the phylum level, Chloroflexi, Cyanobacteria, Desulfobacterota, and Verrucomicrobiota were enriched in T1, whereas Planctomycetota was enriched in T3 (Fig. 2a). At the genus level, *Flexilinea*, *Streptococcus*, *Butyrivibrio*, *Selenomonas*, *Kandleria*, *Anaerovibrio*, *Saccharofermentans*, *Blautia*, *Lachnobacterium*, *Veillonellaceae* *UCG-001*, *Monoglobus*, *Anaerovorax*, *Colidextribacter*, *Lachnospiraceae* *UCG-008*, and *Marvinbryantia* were more abundant in the T1 group than in the other groups, whereas *Ruminococcaceae* *UCG-001*, *Erysipelotrichaceae* *UCG-002*, *Bifidobacterium*, *Shuttleworth*, *Sharpea*, and *Erysipelatoclostridiaceae* *UCG-004* were more enriched in the middle fattening state (T2) group than in the other groups. *Acetitomaculum*, *Erysipelotrichaceae* *UCG-009*, *Mycoplasma*, *Atopobium*, *Rikenellaceae* *SP3-e08*, and *Howardella* were more abundant in the T3 group than in the other groups (Fig. 2b).

### Diversity analysis

**Figure 3:**
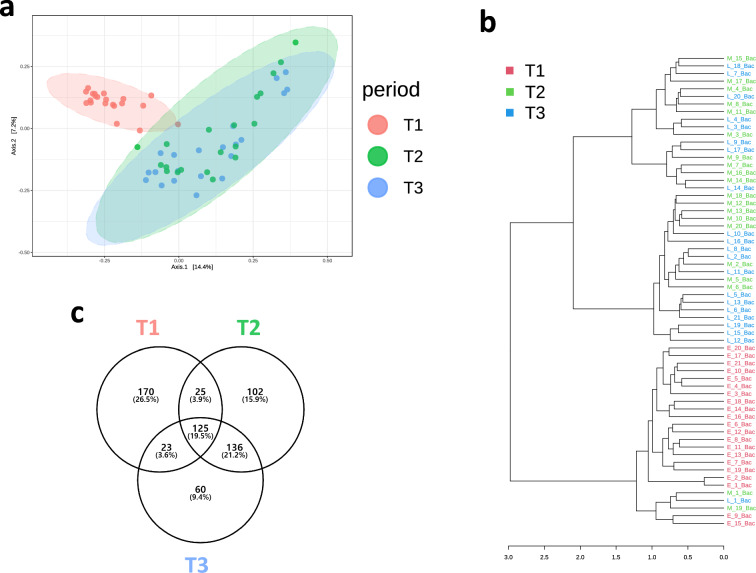
Beta-diversity analysis, hierarchical relationship, and Venn diagram visualization among the three fattening phases. (**a**) PCoA plots based on the Bray–Curtis dissimilarity matrix showing a comparison of the ASVs in the rumen microbes (R-squared = 0.132, P < 0.001). (**b**) Visualized hierarchical relationship. (**c**) Venn diagrams showing the ASVs (relative abundance > 0.05% and prevalence > 20%) of rumen microbiota shared among the three fattening groups. *T1* early fattening period, *T2* middle fattening period, *T3* late fattening period.

**Figure 4:**
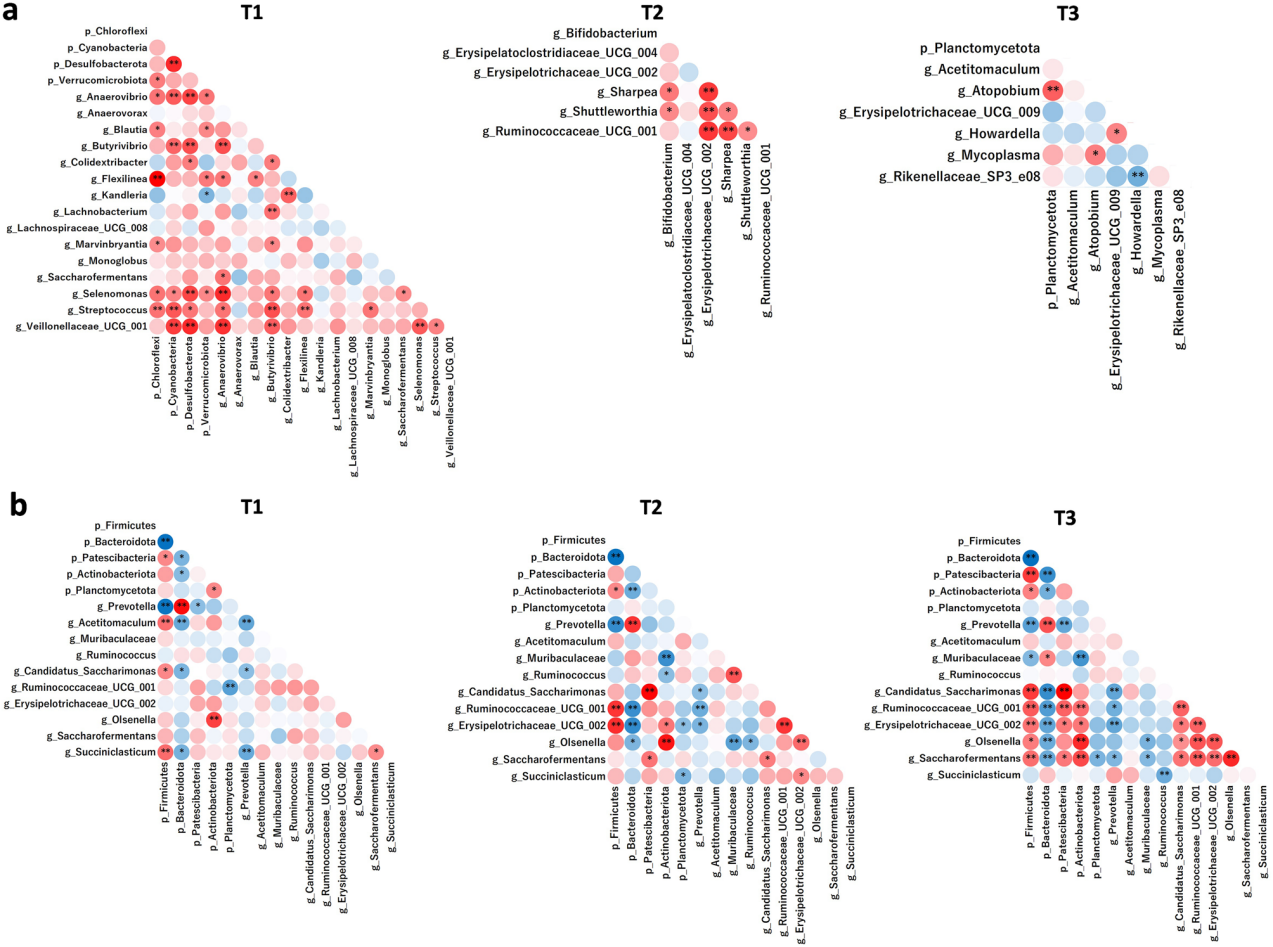
Co-occurrence of ruminal phyla and genera selected using LEfSe (**a**) and major top 5 phyla and top 10 genera (**b**) in each fattening period. Spearman’s correlation represents a positive relationship with red circles and a negative relationship with blue circles. ** and * indicate P < 0.01 and P < 0.05, respectively. *T1* early fattening period, *T2* middle fattening period, *T3* late fattening period.

Alpha diversity analysis revealed no significant differences in the observed amplicon sequence variant (ASVs), Chao1, ACE, and Shannon indices across the three fattening phases (Table 1). However, the observed genera and species indices were significantly higher in the T1 group than in the T2 and T3 groups (P < 0.05). Beta-diversity analysis and hierarchical relationship analysis revealed a significant difference in the overall rumen microbiota composition among the three fattening periods (P < 0.05; Fig. 3a, b). To determine the shared and exclusive ASVs among the fattening periods, a comparative analysis was conducted using Venn diagrams (Fig. 3c). The analysis revealed that out of the 641 ASVs detected, 25, 136, and 23 ASVs were shared among T1–T2, T2–T3, and T3–T1, respectively. Moreover, 170, 102, and 60 ASVs were exclusively detected during the T1, T2, and T3 fattening periods, respectively, whereas 125 ASVs were shared among the three fattening periods. The co-occurrence of ruminal phyla and genera selected using LEfSe and the major top 5 phyla and top 10 genera were analyzed (Fig. 4). In total, 64, 36, and 49 correlations were exclusively detected during the T1, T2, and T3 fattening periods, respectively.

**Table 1:** α-Diversity indices in the three fattening period groups.

Measurements	Fattening period			SEM	P-value
	T1	T2	T3		
Observed ASVs	664.52	682.55	597.38	17.608	0.131
Observed genera	128.2a	121.2ab	115.0b	1.529	< 0.01
Observed species	194.7a	193.0ab	175.8b	2.780	< 0.05
Chao1 estimates	674.97	698.26	608.20	18.252	0.130
ACE	196.83	196.41	178.06	2.848	0.131
Fisher	29.20	28.93	25.92	0.487	< 0.1
Shannon	3.65	3.70	3.58	0.040	0.306
Simpson	0.92	0.94	0.93	0.006	0.679

*T1* early fattening period, *T2* middle fattening period, *T3* late fattening period.

a, b: Means in a row followed by different letters indicates a significant difference (P < 0.05).

### Correlation between physiological parameters and rumen microbiota

**Figure 5:**
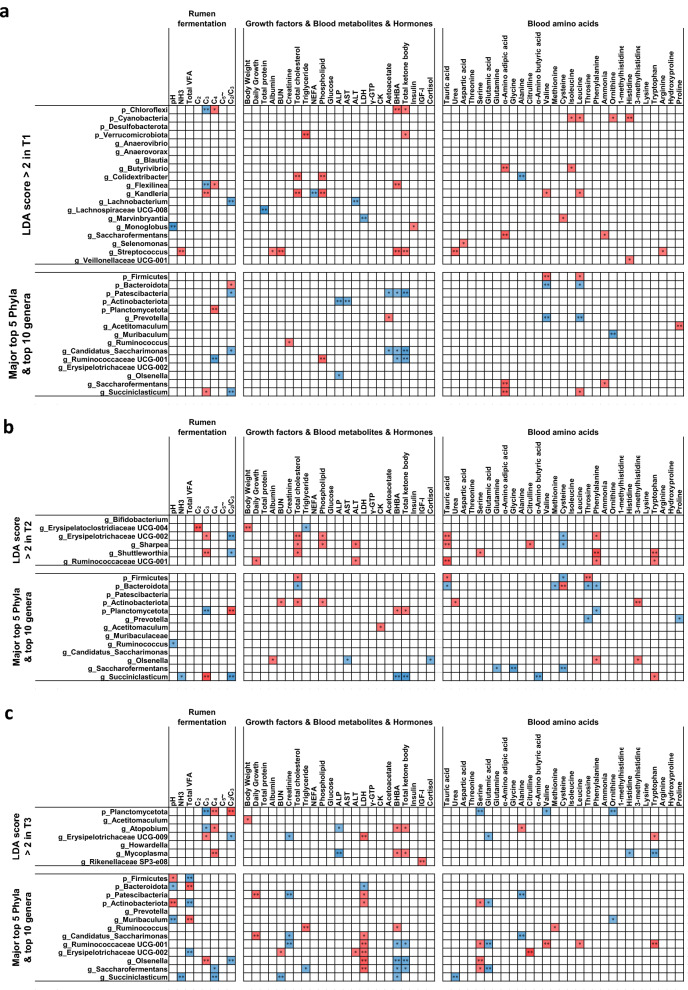
Correlation between physiological parameters and LEfSe-selected and major rumen microbiota. Only strong Spearman’s correlation coefficients (correlation coefficient, |r|≥ 0.5; P < 0.05) are shown (** and * indicate P < 0.01 and P < 0.05, respectively). T1 (**a**), T2 (**b**), and T3 (**c**). The correlation coefficients were based on the intensity of the color. Red and blue color indicate positive and negative correlation coefficients, respectively. *p_* phyla, *g_* genera, *T1* early fattening period, *T2* middle fattening period, *T3* late fattening period, *C*_*2*_ acetic acid, *C*_*3*_ propionic acid, *C*_*4*_ butyric acid, *BUN* blood urea nitrogen, *NEFA* non-esterified fatty acid, *ALP* alkaline phosphatase, *AST* aspartate aminotransferase, *ALT* alanine aminotransferase, *LDH* lactate dehydrogenase, *γ-GTP* gamma(γ)-glutamyl transferase, *CK* creatine kinase, *BHBA* β-hydroxybutyric acid, *IGF-I* insulin-like growth factor 1.

Spearman's correlations were used to assess the relationships between rumen fermentation, growth factors, blood metabolites, hormones, and amino acids and LEfSe-selected and major rumen microbiota (Fig. 5). The results revealed 75, 66, and 82 significant correlations (correlation coefficient, |r|≥ 0.5; P < 0.05) in T1, T2, and T3, respectively. *Streptococcus* showed a positive correlation with ruminal ammonia, blood urea nitrogen (BUN), blood urea concentration, beta-hydroxybutyrate (BHBA), and total ketone body in T1. Body weight showed a positive correlation with *Erysipelatoclostridiaceae* *UCG-004* in T2 and *Acetitomaculum* in T3. Daily growth showed a positive correlation with *Ruminococcaceae* *UCG-001* in T2 and Patescibacteria and *Candidatus* Saccharofermentans in T3. Planctomycetota showed varying correlations with the composition of volatile fatty acids (VFA) throughout the fattening period. Patescibacteria exhibited a positive correlation with daily growth in T3. Firmicutes were positively correlated with pH and negatively correlated with total VFA in T3, whereas Bacteroidota was negatively correlated with pH and positively correlated with total VFA in T3. The correlation between Firmicutes-to-Bacteroidetes (F/B) ratio and physiological parameters is shown in Supplementary Fig. 2. F/B ratio showed a positive correlation with daily growth, ruminal pH, and LDH but showed a negative correlation with total VFA in T3. F/B ratio also showed a positive correlation with total blood cholesterol and phospholipid levels in T2.

### Functional genetic profiles

Functional genetic profiles revealed 8, 5, and 17 differentially abundant MetaCyc pathways (LDA score > 3, P < 0.01) in T1, T2, and T3, respectively (Supplementary Table 3). The MetaCyc pathways associated with nucleoside and nucleotide biosynthesis (PWY-6277, PWY-6122, PWY-7229, PWY-7219, PWY-7221, PWY-5686, and PWY-7208) and several amino acid biosynthesis (PWY-5104, PWY-2942, PWY-5103, and BRANCHED-CHAIN-AA-SYN-PWY) were more abundant in the T3 group than in the other groups. Conversely, the MetaCyc pathways linked to carbohydrates, sugar, cell wall, and aerobic respiration (PWY-7315, LACTOSECAT-PWY, GLUCOSE1PMETAB-PWY, PWY-6470, HEXITOLDEGSUPER-PWY, and PWY-3781) were more prevalent in the T1 group than in the other groups. Additionally, the MetaCyc pathway associated with pyruvate fermentation (PWY-5100) and the two amino acid biosynthesis pathways (PWY-6151 and COMPLETE-ARO-PWY) were more abundant in the T2 group than in the other groups.

## Discussion

We conducted a comprehensive analysis on the rumen microbiome of Japanese Black steers during the fattening phase. Fiber-degrading bacteria and distinct microbial composition were more prevalent in T1, characterized by high forage intake, than in T2 and T3. In T2, an abrupt increase in the proportion of concentrate feed led to varying degrees of adaptation to new feeds, which resulted in distinct microbial clusters among individual cattle. In T3, there was an increase in nucleotides biosynthesis, and F/B ratio, known to be associated with feed efficiency, correlated with growth factors in T3.

Rumen bacterial microbiota underwent significant changes from T1 to T3. Principal coordinate analysis (PCoA) revealed that the rumen microbial composition in T1 was distinct from that in T2 and T3. Cattle were fed a larger amount of forage feed in T1 than in T2 and T3; however, fattening was promoted in T2 and T3 owing to a considerable amount of concentrated feed provided. More forage intake reduces intra-group microbial differences^14^. Furthermore, non-metric multidimensional scaling (NMDS) plots for rumen microbial data from the middle fattening stage showed a more scattered pattern than those from the early fattening stage in previous study of Japanese Black cattle^15^. In the present study, PCoA and dendrogram graphs showed that there was a minimal spatial distance within the intra-group during the T1 period, whereas T2 and T3 exhibited significant spatial distance and mixed distribution. The ASV Venn diagram indicated that T2 and T3 shared 55% ASVs, suggesting that the changes in rumen microbiota were more pronounced from T1 to T2 than from T2 to T3, which could be attributed to changes in feed composition.

The bacterial diversity in the rumen decreases as the forage-to-concentrate ratio of feed decreases^16^. Compared to high-forage feed, high-concentrate feed reduced the variety of bacteria present in the rumen, owing to a decrease in pH^17^. The number of ASVs found exclusively in T1 (170 ASVs) was higher than that found in T2 (102 ASVs) or T3 (60 ASVs). Similarly, alpha diversity analysis showed that T1 had a more diverse microbial community than T2 and T3, and this is believed to be due to the decrease in the number of fiber-decomposing bacteria caused by the decrease in forage feed ratio. In agreement with the decrease in alpha diversity, the absolute quantities of total bacteria, ciliate protozoa, and fungi decreased during transition from early to middle and late fattening stages. This result aligns with data indicating a decrease in microbial diversity observed from the early to late fattening stages in Japanese Black cattle^15^. As the body grew, feed intake, rumen volume, and rumen fluid volume increased in the late fattening stage compared with those in the early fattening stage; however, the rumen fluid in the early fattening period contained a greater diversity and higher concentration of microorganisms than that in the late fattening stage.

LefSe analysis revealed 15, 6, and 6 differentially abundant genera in T1, T2, and T3, respectively. Flexilinea, a fiber-digesting bacterium that degrades all types of carbohydrates^18^, was shown to have a significantly higher relative abundance in the group fed low-dietary energy feed than in the high-dietary energy group^19^. Although the function of Flexilinea in the rumen has not been revealed, our study showed that its abundance was higher in T1, with a relatively low-dietary energy feed. Chloroflexi has been reported to be associated with hydrolysis of cellulose^20^, and its abundance was higher in T1 fed a relatively high forage than in T2 and T3. Chloroflexi and *Flexilinea*, which are fiber-degradable bacteria, were positively correlated with ruminal butyric acid concentration and negatively correlated with propionic acid concentration in T1. Some bacteria involved in digesting fiber may function to generate butyrate^21^, and high-concentrate diet may increase the production of propionic acid in the rumen^22^. Although the function of Chloroflexi and *Flexilinea* was not fully elucidated, these bacteria may have been responsible for the higher butyric acid and lower propionic acid concentration in T1 than in T2 and T3. Butyrate is a ketogenic VFA that undergoes metabolic conversion to BHBA when it is being absorbed through the rumen epithelium^23^. There were positive correlations between these bacteria and BHBA. These correlations could potentially be attributed to the fiber-degrading ability of these bacteria, rather than indicating a direct relationship between these bacteria and BHBA. *Butyrivibrio* is one of the main butyrate producers and plays a role in developing tight junctions in the rumen epithelial layer^24^. The relative percentages of *Butyrivibrio* and butyric acid concentrations in the rumen were higher in T1 than in T2 and T3 and might affect the growth of epithelial cells*.*

*Streptococcus*, a ureolytic bacterium, was more abundant in T1 than in T2 and T3, and it showed a positive correlation with ruminal ammonia and BUN in T1. *Streptococcus* becomes more abundant in response to urea supplementation and is responsible for producing urease and breaking down urea into ammonia^25^. BUN, which is synthesized in the liver and transported to the rumen, is a source of nitrogen for microbial protein synthesis^26^. Diets rich in highly soluble crude proteins can increase BUN^27^. The higher protein intake in T1 may explain the stronger correlation among *Streptococcus*, NH_3_, and BUN, compared with that in T2 and T3. *Marvinbryantia*, which has the ability to degrade cellulose^28^, was more abundant in T1 fed higher forage feed than in T2 and T3. *Blautia*, which produces acetic acid and butyric acid^29^, were more abundant in T1 than in T2 and T3 and may contribute to the higher rumen acetate and butyrate concentrations in T1 than in T2 and T3. *Monoglobus* was reported to ferment dietary fiber and its ability to digest complex polysaccharides potentially facilitating butyrate production^30^. *Monoglobus* was more abundant in T1 fed higher forage feed than T2 and T3 and may contribute to the higher rumen concentration of butyrate in T1 than in T2 and T3. Butyric acid led to a decrease the rumen pH^31^. Although Monoglobus did not show a correlation with butyric acid, the negative correlation between this genus and ruminal pH in T2 could explained by the ability of this genus to produce butyrate. *Acetitomaculum* mainly occurs in ruminants that feed on a high-concentrate diet^32^ and can generate acetate from monosaccharides^33^. The higher ruminal acetate in T3, during which animals were fed a high-concentrate diet, could be due to the relatively higher abundance of acetate producers such as *Acetitomaculum*. Additionally, owing to the positive correlation observed between this genus and body weight, it is important to focus on its potential influence on growth during T3. *Mycoplasma* has been associated with various diseases in cattle, including arthritis, pneumonia, mastitis, otitis media, and reproductive disorders^34^. Although research on the impact of *Mycoplasma* on rumen diseases is limited, it is important to note its potential effect during the late fattening period, when various diseases such as acidosis may arise due to high-concentrate feed. *Erysipelatoclostridiaceae* is related to obesity in human gut study^35^, and *Erysipelatoclostridium* are abundant in chickens with increased body weight^36^. A study in mice revealed that *Erysipelatoclostridium* was positively correlated with body weight and fat deposition^37^. In this study, *Erysipelatoclostridiaceae* UGC-004 demonstrated a positive correlation with acetic acid and body weight, suggesting the potential impact of this bacteria on animal growth. Proteobacteria is reportedly the third most dominant phylum in the rumen of the different cattle species^38–40^. However, in this study, Patescibacteria (2.73%) was the third most dominant phylum, whereas Proteobacteria (0.26%) was the eighth most dominant phylum. Patescibacteria are commonly present in anaerobic environments, such as sediments, groundwater, and various other oxygen-deprived habitats^41^. Although the functions of this microorganism are not well elucidated^42^, the present study indicates a positive relationship between this phylum and daily growth in T3. This finding underscores the importance of conducting an additional study on this microorganism, suggesting that it may play a crucial role in growth during late fattening periods. Planctomycetes exhibited varied correlations with rumen VFAs. It had a positive correlation with butyrate in T1 and T3 and acetate/propionate ratio (C_2_/C_3_) in T2 and T3 but showed a negative correlation with propionate in T2 and T3. Interestingly, this phylum reportedly has a significant positive correlation with valerate in the rumen^43^. A study on Angus cattle revealed that Planctomycetes were more abundant in grass-fed cattle, which had higher levels of VFAs in their rumen^44^. Additionally, identification of the enzymes responsible for C_1_ metabolism in Planctomycetes has contributed to a better understanding of methylotrophic and methanogenesis pathways^45^. The formation of CH_4_ during rumen fermentation is closely associated with the profile of VFAs^46^. Although there is no direct evidence linking Planctomycetota to VFA production in the rumen, this phylum may influence the composition of VFAs in Japanese Black cattle.

Intramuscular fat deposition occurs during the late fattening period of beef cattle^47^. Studies have shown a positive correlation between the F/B ratio and average daily gain in ruminants^48,49^. In the present study, the F/B ratio in T3 showed a positive correlation with daily gain. Additionally, the F/B ratio in gut microbiota can promote fat storage^50^, and it is associated with obesity owing to the production of more enzymes involved in energy production and storage with an increase in the F/B ratio^51^. Moreover, the F/B ratio in the rumen has a positive correlation with milk fat yield in cows^52,53^. In this study, we focused on the F/B ratio as an indicator of fat deposition during the fattening period. The correlation between the F/B ratio and daily gain in T3 might be attributed to the higher weight gain and fat deposition that occur during the late fattening period when the feed is more concentrated. The F/B ratio also exhibited a positive correlation with LDH levels in T3, supporting the link between the F/B ratio and daily gain due to fat deposition. LDH plays a crucial role in regulating gluconeogenesis^54^. Ruminants primarily depend on glucose produced through gluconeogenesis for energy^55^, as excess glucose is converted into fatty acids and stored in adipose tissue^56^. Increased liver gluconeogenesis is linked to elevated concentrations of blood lipid metabolites during the late fattening period^8^. The consumption of high-energy diets in T3 may have resulted in increased LDH and gluconeogenesis and subsequent conversion into fat deposition. Although there is insufficient evidence to explain the correlation between LDH and the F/B ratio, these two factors may be correlated to fat accumulation. This needs further investigation in the future. The F/B ratio had a negative correlation with total VFA concentration in T3 and positive correlation with rumen pH in T3. These results are consistent with those of a previous report that suggested that a decline in the F/B ratio was associated with a lower rumen pH and an increased accumulation of VFAs at high concentrations of organic substrates^57,58^. VFAs promote fat oxidation and energy expenditure while decreasing lipolysis, thus reducing the risk of obesity^59^. Individual cattle with low VFA production in T3 may exhibit an increased F/B ratio and fat accumulation. Although the F/B ratio did not show a correlation with daily gain in T2, the positive correlation between F/B ratio and blood total cholesterol and phospholipids in T2 suggests that F/B ratio may also serve as an indicator of fat accumulation in T2.

Nucleotides provide many health benefits to humans^60^ and have been included in infant formulas for several years^61^; similar advantages have been observed in livestock^62,63^. In ruminants, nucleotides improve the development of the gastrointestinal tract^62^, enhance animal performance^64^, and prevent neonatal diarrhea^62^. Nucleotide supplementation reportedly decreases the incidence of respiratory problems in dairy calves during the weaning period^65^. In the present study, the functional genetic profiles of the rumen microbiome indicated an increase in nucleoside and nucleotide biosynthesis in T3. This may explain the upregulation in energy metabolism and ATP synthesis, such as in PWY-7229 and PWY-7219, promoted by high-energy feed in T3. Additionally, cattle fed on diets supplemented with nucleotides had a higher rumen pH than the group that did not receive nucleotide supplementation^66^. Although research on the relationship between nucleotides and rumen environment is limited, nucleotides may help to maintain a stable ruminal pH and prevent subacute ruminal acidosis in T3.

## Conclusion

Our study revealed significant physiological changes in Japanese Black steers during different fattening phases, primarily influenced by feed variations. Rumen microbial diversity and quantity were notably higher in T1, with a composition distinct from that of T2 and T3. Specific genera showed increased abundances in T1 (*Flexilinea*, *Streptococcus*, *Butyrivibrio*, *Selenomonas*, and *Kandleria*) and T3 (*Acetitomaculum*, *Mycoplasma*, *Atopobium*, and *Howardella*). Specific microorganisms are associated with certain physiological parameters. The F/B ratio was positively correlated with daily gain and LDH in T3. Planctomycetota showed correlation with VFA compositions, and *Streptococcus* was associated with ammonia metabolism. These findings suggest that the correlation between physiological characteristics and ruminal microbiota can serve as a criterion for assessing the metabolic status of Japanese Black cattle. Furthermore, this information could facilitate the development of strategies to enhance rumen health and productivity by manipulating rumen microbiota through suitable feeds, considering the fattening stages of Japanese Black cattle.

## Method

### Animals and rumen sample collection

All animal experiments were conducted at the Hyogo Prefectural Technology Center of Agriculture, Forestry, and Fisheries (Hyogo Prefecture, Japan), following the ethical guidelines of the Institute of Livestock and Grassland Science^67^ and the Animal Care and Use Committee of Hyogo Prefectural Institute of Agriculture, Forestry, and Fisheries. The experimental procedure was approved by the Animal Care and Use Committee of the Hyogo Prefectural Institute of Agriculture, Forestry, and Fisheries (approval number: H2018-01). All animal studies followed the ARRIVE guidelines (https://arriveguidelines.org).

A total of 21 Japanese Black cattle were used to evaluate the composition of rumen microbiota according to their fattening stages. The experimental animals were reared from 12 months of age (initial body weight, 335.6 ± 19.8 kg) until 30 months of age (final body weight, 742.1 ± 49.9 kg) and were separated into three groups based on the fattening phase: early fattening (12–14 months of age; T1), middle fattening (15–22 months of age; T2), and late fattening phases (23–30 months of age; T3). Feeding management followed the procedures of the Hyogo Prefectural Technology Center of Agriculture, Forestry, and Fisheries, and the experimental animals were fed a specified formula feed, which was adjusted throughout each fattening period. Blood and ruminal fluid samples were collected at each fattening period (13, 20, and 28 months of age). Blood samples were collected from the jugular vein into heparin-sodium tubes (Venoject II VP-H100K, Terumo, Tokyo, Japan). Rumen fluid was obtained via a catheter, and approximately 200 mL fluid was collected in a sterilized and dried flask. Comparison of rumen fermentation characteristics, blood metabolites, hormones, and blood amino acids in each fattening phase was reported in our previous paper^8^. The nutrient composition of formulated diets, amount of feed intake and growth performance were shown in Supplementary Table 4.

### DNA extraction and amplicon sequencing

DNA was extracted from rumen fluid using a MP Biomedicals Fast DNA kit following the manufacturer's protocol for the amplification of 16S rRNA genes. Genomic DNA was quantified using the Nanodrop ND-1000 Spectrophotometer and agarose gel electrophoresis was performed to verify its integrity. One sample (M_19) from T2 was excluded because of poor gDNA quality. Bacterial 16S rRNA V3-V4 region libraries were generated using Illumina’s 16S metagenomics sequencing library preparation protocol and the Herculase II Fusion DNA Polymerase Nextera XT Index V2 Kit. Thereafter, sequencing was conducted on the Illumina MiSeq platform (San Diego, CA, USA) using the 2 × 300 bp paired-end method. Information on the selected primers targeting the bacterial 16S rRNA gene is provided in Supplementary Table 2.

### Metagenomic profiles and functional genetic analyses

The 16S amplicon sequence reads obtained were analyzed using QIIME2 version 2023.02 (http://qiime.org/)^68^. The adapter and primer sequences were trimmed using Cutadapt^69^. Chimeric sequences were removed. Denoising, quality filtering (Q-score > 25), and merging were conducted using the plugin DADA2^70^ to create the ASV feature table. The taxonomic classifiers were manually trained using the Naive Bayes classifier^71^, with the SILVA version 138 database^72^ clustered at 99% similarity. The taxonomic assignment of ASVs was performed using the plugin q2-feature-classifier with 99% bacterial identity and representative sequences. Following the first taxonomic classification, an extra filtration step was used to obtain bacterial sequence data. Unassigned ASVs, chloroplasts, mitochondria, and non-bacterial taxa were excluded from taxonomic filtration.

The alpha and beta diversities of the overall rumen microbiota in T1, T2, and T3 were analyzed using MicrobiomeAnalyst^73^ based on the ASV biological observation matrix (BIOM). Alpha diversity indices, including species richness (Chao1, ACE-observed ASVs, observed genera, and observed species), and Fisher, Shannon, and Simpson's indices were analyzed. The differences in bacterial composition were represented using PCoA based on the Bray–Curtis dissimilarity matrix^74^, and the hierarchical relationship was visualized using a dendrogram. To compare the number of shared and exclusive ASVs in each group, a Venn diagram was generated using the Venny software version 2.1 (https://bioinfogp.cnb.csic.es/tools/venny/)^75^.

Functional genetic profiles were predicted u`sing Phylogenetic Investigation of Communities by Reconstruction of Unobserved States 2 (PICRUSt2) version 2.5.1^76^. The percentage relative abundance counts of the MetaCyc profiles were used to predict the overall functional differences among the three fattening stages.

### Absolute quantification of rumen microbiome in real-time PCR

Gene copies of total bacteria, fungi, and ciliate protozoa were determined through quantitative real-time PCR using the TB Green™ Premix Ex Taq™ II (Takara Biomedical Technology). The triplicate PCR quantification was performed with the reaction mixture consisting of 5 μL of TB Green Premix Ex Taq II, 1 μL of gDNA (10 ng/μL), 0.5 μL each of primer-set, and 3 μL of Diethylpyrocarbonate (DEPC). The thermal cycling parameters were as follows: 95 °C for 15 min, followed by 30 cycles at 98℃ for 10 s, 55 °C for 15 s, and 72 °C for 60 s. For the standard curve, qPCR quantification was performed under the same conditions using primeSTAR^®^ HS DNA (Takara Biomedical Technology), and the reaction mixture consisted of 2 μL of 2.5 mM dNTP mix, 8 μL of 5× PrimeSTAR Buffer, 1 μL of gDNA (10 ng/μL), 0.5 μL each of primer-set, 0.5 μL of PrimeSTAR HS DNA polymerase, and 27.5 μL of DEPC. The PCR products were purified using the Gel/PCR Extraction Kit (FastGene), and qRT-PCR was conducted under the same conditions. To establish five standard reference points, a series of tenfold dilutions was performed, and copy numbers were calculated as described by Chen and Oba^77^. The primers used for quantitative real-time PCR are listed in Supplementary Table 2.

### Statistical analysis

Statistical differences in alpha diversity and absolute quantification of rumen microbiome were evaluated among the three fattening stages using analysis of variance with R (v.4.1.3) software. Spearman's correlation was performed to analyze the correlation coefficients between physiological parameters and rumen microbiota for each fattening period. All the physiological parameter data on growth factors, rumen fermentation characteristics, blood metabolites, and amino acids used in the correlation analysis were obtained from our previous study^2^.

The reads in each ASV were normalized to their relative abundance in each sample. Differential microbial taxa comparisons were conducted among different fattening stages, and differential functional genetic profiles were identified using LEfSe^78^, with a logarithmic LDA score of 2 as the cutoff. Only the taxa with a relative abundance greater than 0.05% and a prevalence of at least 20% were used for the LEfSe and Venn diagram. Taxa that were poorly identified and had a prevalence of < 90% were excluded from the correlation analysis. Cladograms were plotted using the Galaxy Hutlab web. Permutational multivariate analysis of variance was used to conduct beta-diversity analysis among the three fattening groups, using PAST3 with 9999 random permutations^79^. Co-occurrence relationships of the microbiota were analyzed using the R (v.4.1.3) software package "Hmisc.” Statistical differences were declared at P ≤ 0.05, and statistical tendencies were declared at 0.05 < P ≤ 0.10.

### Supplementary Information

Supplementary Information.

## Data Availability

Raw sequence reads data obtained from the present study have been deposited in the NCBI BioProject database, assigned the project number PRJNA1031765. The data will be available with the following link: https://www.ncbi.nlm.nih.gov/bioproject/PRJNA1031765.
