# Supplementary material for: Assessing the impact of three feeding stages on rumen bacterial community and physiological characteristics of Japanese Black cattle
Source: Sci Rep. 2024 Feb 28;14:4923. doi: 10.1038/s41598-024-55539-y (PMC10902337; doi:10.1038/s41598-024-55539-y)
Supplement: Supplementary file 1 — Supplementary Information. [file 41598_2024_55539_MOESM1_ESM.pdf]

Assessing the impact of three feeding stages on rumen bacterial community and physiological characteristics of Japanese Black cattle

Huseong Lee[1], Minji Kim[2], Tatsunori Masaki[3], Kentaro Ikuta[3], Eiji Iwamoto[3], Koki Nishihara[1], Itoko Nonaka[2], Akane Ashihara[2], Youlchang Baek[4], Sungdae Lee[4], Yoshinobu Uemoto[1], Satoshi Haga[1], Fuminori Terada[2], and Sanggun Roh[1*]

[1] Graduate School of Agricultural Science, Tohoku University, Sendai 980-8572, Japan
[2] National Institute of Livestock and Grassland Science, National Agriculture and Food Research Organization, Ikenodai, Tsukuba 305-0901, Japan
[3] Hyogo Prefectural Technology Center of Agriculture, Forestry and Fisheries, Kasai, Hyogo 679-0198, Japan
[4] Animal Nutrition & Physiology Division, National Institute of Animal Science, Wanju 55365, South Korea
*sanggun.roh@tohoku.ac.jp

Supplementary legends

Supplementary table 1. Overview of Illumina MiSeq sequenced datasets.

	T1	T2	T3
No. of input paired reads	117,455 ± 22,391	132,030 ± 32,677	105,921 ± 18,536
Quality-filtered sequences	91,778 ± 17,312	104,579 ± 26,808	83,629 ± 15,383
Percentage of filter sequences (%)	78.2 ± 2.3	79.1 ± 1.9	78.9 ± 2.0
Denoised sequences	83,811 ± 16,933	96,936 ± 25,773	77,934 ± 14,456
Merged sequences	621,99 ± 14,597	74,130 ± 21,040	60,999 ± 11,522
Percentage of denoised and merged sequences (%)	52.7 ± 5.0	55.8 ± 4.0	57.6 ± 3.6
Non-chimeric sequences	42,546 ± 10,234	50,958 ± 15,012	44,434 ± 9,107
Percentage of non-chimeric sequences (%)	36.1 ± 5.2	38.4 ± 5.4	42.4 ± 7.3

T1, early fattening period; T2, middle fattening period; T3, late fattening period.

Supplementary table 2. Information on primers used for MiSeq Illumina sequencing and q-RT-PCR.

Target	Primer sequence (5′ → 3′)	Amplicon size (bp)	Usage	References
Quantitative real-time PCR				
Total bacteria	F: CGGCAACGAGCGCAACCC<br>R: CCATTGTAGCACGTGTGTAGCC	130	qPCR	[1]
Ciliate protozoa	F: GCTTTCGWTGGTAGTGTATT<br>R: CTTGCCCTCYAATCGTWCT	223	qPCR	[2]
Fungi	F:GAGGAAGTAAAAGTCGTAACAAGGTTTC<br>R: CAAATTCACAAAGGGTAGGATGATT	120	qPCR	[1]
Amplicon sequencing				
Bacterial 16S rRNA (341F-806R)	F:CCTACGGGNGGCWGCAG<br>R:GACTACHVGGGTATCTAATCC	530	Sequencing	[3]

1. Denman, S. E. & McSweeney, C. S. Development of a real-time PCR assay for monitoring anaerobic fungal and cellulolytic bacterial populations within the rumen. *FEMS Microbiol. Ecol.* **58**, 572-582; https://doi.org/10.1111/j.1574-6941.2006.00190.x (2006).
2. Sylvester, J. T., Karnati, S. K., Yu, Z., Morrison, M. & Firkins, J. L. Development of an assay to quantify rumen ciliate protozoal biomass in cows using real-time PCR. *J. Nutr.* **134**, 3378-3384; https://doi.org/10.1093/jn/134.12.3378 (2004).
3. PCR Amplicon., PCR Clean-Up & PCR Index. 16S Metagenomic sequencing library preparation. Illumina: San Diego, CA, USA (2013).

Supplementary table 3. Differentially abundant MetaCyc pathway profiles among the three fattening phases.

**MetaCyc pathway**	Class	LDA score	P-value	**Description**	**Superclasses**
PWY-6263	T1	3.16	0.002	superpathway of menaquinol-8 biosynthesis II	Biosynthesis
PWY-7315	T1	3.25	0.004	dTDP-N-acetylthomosamine biosynthesis	Carbohydrates Biosynthesis
LACTOSECAT-PWY	T1	3.08	<0.001	lactose and galactose degradation I	Carbohydrates Degradation
GLUCOSE1PMETAB-PWY	T1	3.06	<0.001	glucose and glucose-1-phosphate degradation	Carbohydrates Degradation
PWY-6470	T1	3.12	<0.001	peptidoglycan biosynthesis V	Cell Wall Biosynthesis
PWY-3781	T1	3.71	0.009	aerobic respiration I	Generation of Precursor Metabolites and Energy
PWY-6703	T1	3.46	0.005	preQ_0 biosynthesis	Secondary Metabolite Biosynthesis
HEXITOLDEGSUPER-PWY	T1	3.26	0.009	superpathway of hexitol degradation	Sugar Derivatives Degradation
PWY-6151	T2	3.47	0.005	S-adenosyl-L-methionine cycle I	Amino Acids Biosynthesis
COMPLETE-ARO-PWY	T2	3.23	0.006	superpathway of aromatic amino acid biosynthesis	Amino Acids Biosynthesis
PWY-5100	T2	3.48	<0.001	pyruvate fermentation to acetate and lactate II	Pyruvate Fermentation
NONMEVIPP-PWY	T2	3.12	0.002	methylerythritol phosphate pathway I	Secondary Metabolites Biosynthesis
PWY-7560	T2	3.12	0.002	methylerythritol phosphate pathway II	Secondary Metabolites Biosynthesis
PWY-5104	T3	3.36	0.005	L-isoleucine biosynthesis IV	Amino Acid Biosynthesis
PWY-2942	T3	3.26	0.000	L-lysine biosynthesis III	Amino Acid Biosynthesis
PWY-5103	T3	3.12	0.003	L-isoleucine biosynthesis III	Amino Acid Biosynthesis
BRANCHED-CHAIN-AA-SYN-PWY	T3	3.11	0.001	superpathway of branched amino acid biosynthesis	Amino Acid Biosynthesis

PWY-6163	T3	3.19	0.001	chorismate biosynthesis from 3-dehydroquinate	Aromatic Compounds Biosynthesis
PWY-5121	T3	3.1	0.003	superpathway of geranylgeranyl diphosphate biosynthesis II	Biosynthesis
PYRIDNUCSYN-PWY	T3	3.26	0.004	NAD biosynthesis I	Cofactors, Prosthetic Groups, Electron Carriers Biosynthesis
PWY-7377	T3	3.27	0.009	cob(II)yrinate a,c-diamide biosynthesis I	Cofactors, Prosthetic Groups, Electron Carriers Biosynthesis
COA-PWY	T3	3.18	0.001	coenzyme A biosynthesis I	Cofactors, Prosthetic Groups, Electron Carriers Biosynthesis
PWY-6167	T3	3.06	<0.001	flavin biosynthesis II	Cofactors, Prosthetic Groups, Electron Carriers Biosynthesis
PWY-5686	T3	3.25	<0.001	UMP biosynthesis	Nucleosides and Nucleotides Biosynthesis
PWY-6277	T3	3.23	0.001	superpathway of 5-aminoimidazole ribonucleotide biosynthesis	Nucleosides and Nucleotides Biosynthesis
PWY-6122	T3	3.23	0.001	5-aminoimidazole ribonucleotide biosynthesis II	Nucleosides and Nucleotides Biosynthesis
PWY-7229	T3	3.17	0.005	superpathway of adenosine nucleotides de novo biosynthesis I	Nucleosides and Nucleotides Biosynthesis
PWY-7219	T3	3.12	<0.001	adenosine ribonucleotides de novo biosynthesis	Nucleosides and Nucleotides Biosynthesis
PWY-7208	T3	3.12	0.004	superpathway of pyrimidine nucleobases salvage	Nucleosides and Nucleotides Biosynthesis
PWY-7221	T3	3.05	0.001	guanosine ribonucleotides de novo biosynthesis	Nucleosides and Nucleotides Biosynthesis

Differential abundance among the three fattening phases was analyzed using linear discriminant analysis (LDA) effect size (LEfSe) (LDA score > 3, P < 0.01). T1, early fattening period; T2, middle fattening period; T3, late fattening period.

Supplementary table 4. Growth performance and nutrient composition of diet at three stages of fattening periods

Variable	Fattening period		
	T1	T2	T3
Feed intake (kg/day)			
Concentrate	5.41 (4.13–5.61)	7.31 (5.96–8.30)	7.44 (4.67–8.96)
Rice straw	1.93 (1.21–2.70)	1.13 (0.72–1.61)	0.74 (0.49–0.99)
Kraft pulp feed	0.72 (0.00–1.54)	0.45 (0.00–0.92)	0.29 (0.00–0.60)
Dry matter	6.98 (5.67–7.27)	7.72 (6.52–8.65)	7.36 (4.89–8.69)
Total digestible nutrients	4.95 (3.94–5.28)	5.95 (5.00–6.69)	5.84 (3.84–6.89)
Crude protein	0.95 (0.74–1.02)	0.93 (0.75–1.07)	0.93 (0.59–1.12)
Ingredient of concentrate (%)			
Steam-flaked corn	42.0	42.0	44.0
Wheat bran	27.0	21.0	14.0
Barley	-	14.0	25.0
Corn gluten feed	10.0	5.0	-
Soybean meal	12.0	10.0	5.0
Soybean hull	6.0	6.0	10.0
Salt	1.0	1.0	1.0
Others	2.0	1.0	1.0
Growth performance			
Body weight (kg)	369.8 (337.7–400.7)	522.6 (478.9–584.3)	686.9 (607.0–757.0)
Average dairy gain (kg/day)	0.86 (0.45–1.07)	0.83 (0.61–1.04)	0.61 (0.49–0.80)

T1, early fattening period; T2, middle fattening period; T3, late fattening period; Concentrate T1: DM 87.3%, TDN 71.2%, CP 15.9%; Concentrate T2: DM 87.2%, TDN 72.5%, CP 14.4%; Concentrate T3: 87.3%, TDN 72.8%, CP 12.0%. Rice straw: DM 87.8%, TDN 37.7%, CP 4.7%. Kraft pulp feed: DM 76.9%, TDN 51.1%, CP 0.3%

Note. Data are from Kim, M. *et al*, 2022 [8]

Supplementary figure 1. Absolute quantification of rumen microbiome in each fattening period.

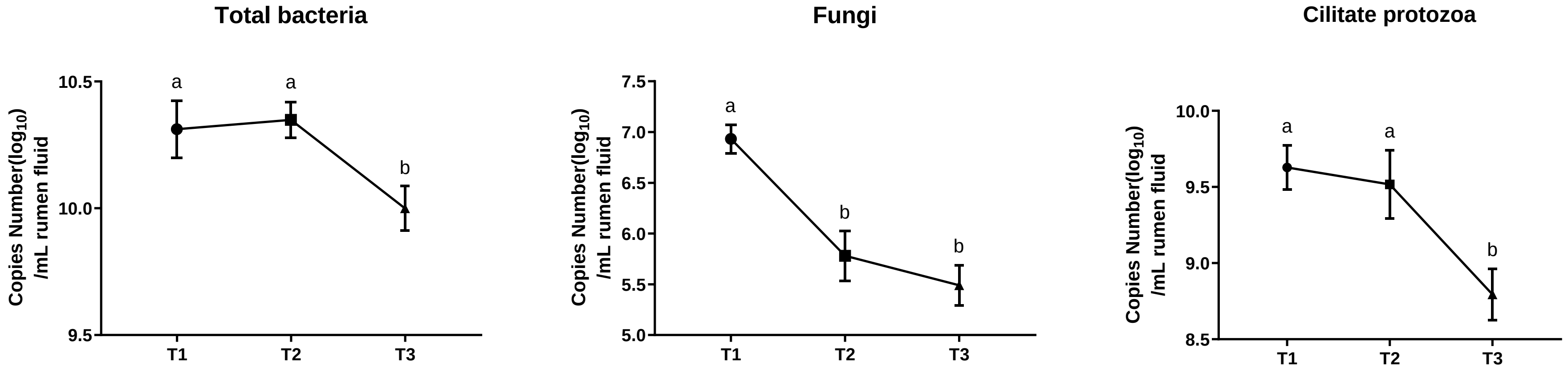

T1, early fattening period; T2, middle fattening period; T3, late fattening period. [a, b] Means in a row followed by different letters indicates a significant difference ($P < 0.05$).

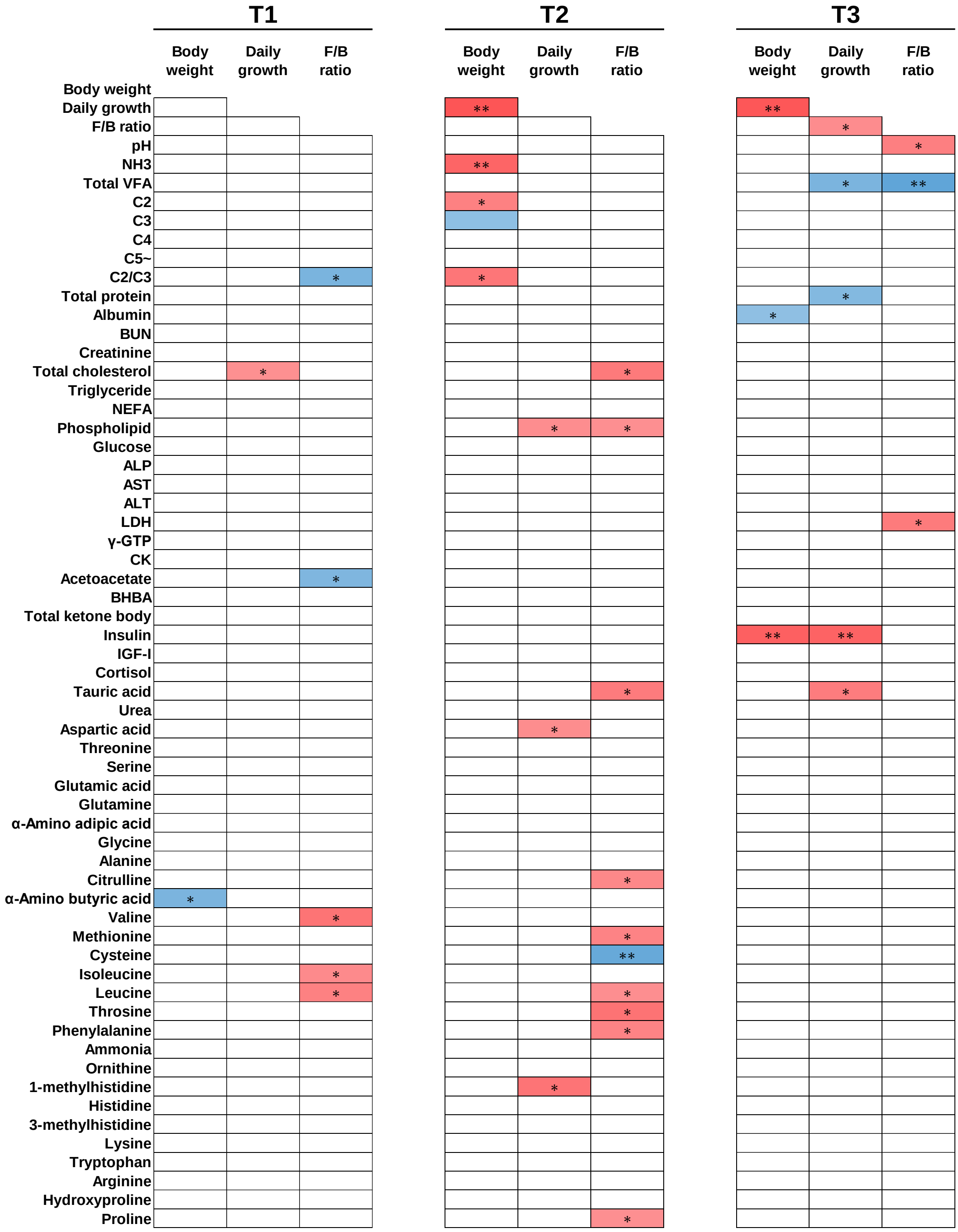

Supplementary figure 2. Correlation among physiological parameters, growth factors, and Firmicutes to Bacteroidota ratio in three fattening periods. Only strong Spearman's correlation coefficients (P ≤ 0.05) are shown on the plot. The correlation coefficients were based on the intensity of the color. The red and blue color indicate positive and negative correlation coefficients, respectively. T1, early fattening period; T2, middle fattening period; T3, late fattening period; C_2, acetic acid; C_3, propionic acid; C_4 butyric acid; BUN, blood urea nitrogen; NEFA, non-esterified fatty acid; ALP, alkaline phosphatase; AST, aspartate aminotransferase; ALT, alanine aminotransferase; LDH, lactate dehydrogenase; γ-GTP, gamma(γ)-glutamyl transferase; CK, creatine kinase; BHBA, β-hydroxybutyric acid; IGF-I, insulin-like growth factor 1.

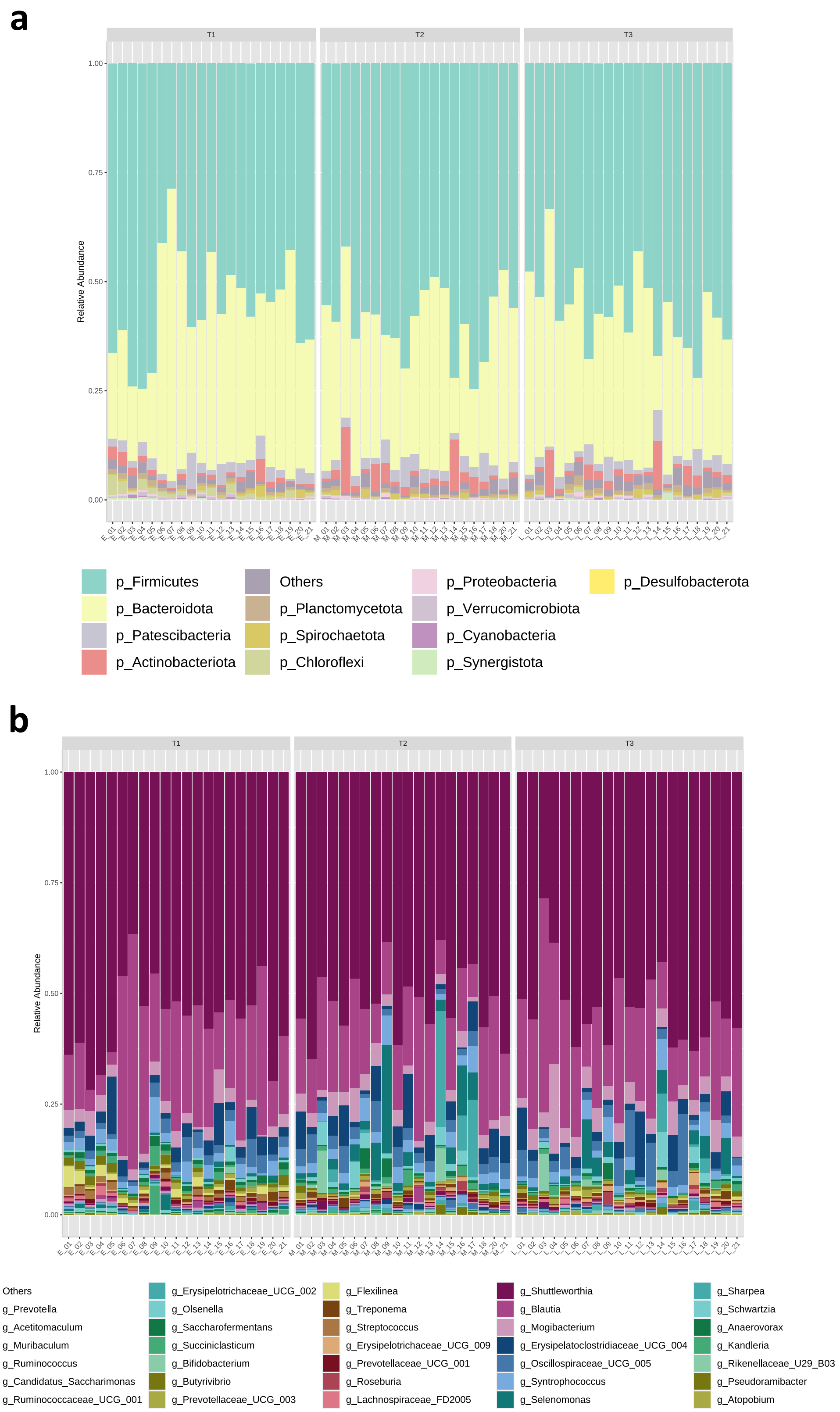

Supplementary figure 3. Composition of rumen microbiota in individual Japanese Black cattle. The relative abundances of (**a**) phyla and (**b**) genera are visualized. Only taxa with a percentage relative abundance of >0.05% and prevalence of at least 20% in 62 Japanese Black cattle are shown and taxa accounting for <0.05% of all sequences and prevalence less than 20% are included in "Others." T1, early fattening period; T2, middle fattening period; T3, late fattening period; p_, phyla; g_, genus.
